# The CRISP colorectal cancer risk prediction tool: an exploratory study using simulated consultations in Australian primary care

**DOI:** 10.1186/s12911-017-0407-7

**Published:** 2017-01-19

**Authors:** Jennifer G Walker, Adrian Bickerstaffe, Nadira Hewabandu, Sanjay Maddumarachchi, James G Dowty, CRECRC, Mark Jenkins, Marie Pirotta, Fiona M Walter, Jon D Emery

**Affiliations:** 1 Centre for Cancer Research, Department of General Practice, VCCC, University of Melbourne, Level 10, 305 Grattan Street, Melbourne, VIC 3010 Australia; 2 Centre for Epidemiology and Biostatistics, Melbourne School of Population and Global Health, University of Melbourne, Melbourne, Australia; 3 Australian NHMRC Centre for Research Excellence: Optimising Colorectal Cancer Screening, Camperdown, Australia; 4 General Practice, School of Primary Aboriginal and Rural Health Care, University of Western Australia, Crawley, WA Australia; 5 The Primary Care Unit, Department of Public Health & Primary Care, University of Cambridge, Cambridge, United Kingdom

**Keywords:** Colorectal cancer, Colorectal cancer risk assessment, Colorectal cancer risk prediction, Primary care, Cancer screening, General practitioners, Bowel cancer screening

## Abstract

**Background:**

In Australia, screening for colorectal cancer (CRC) with colonoscopy is meant to be reserved for people at increased risk, however, currently there is a mismatch between individuals’ risk of CRC and the type of CRC screening they receive.

This paper describes the development and optimisation of a Colorectal cancer RISk Prediction tool (‘CRISP’) for use in primary care. The aim of the CRISP tool is to increase risk-appropriate CRC screening.

**Methods:**

CRISP development was informed by previous experience with developing risk tools for use in primary care and a systematic review of the evidence. A CRISP prototype was used in simulated consultations by general practitioners (GPs) with actors as patients. GPs were interviewed to explore their experience of using CRISP, and practice nurses (PNs) and practice managers (PMs) were interviewed after a demonstration of CRISP. Transcribed interviews and video footage of the ‘consultations’ were qualitatively analyzed. Themes arising from the data were mapped onto Normalization Process Theory (NPT).

**Results:**

Fourteen GPs, nine PNs and six PMs were recruited from 12 clinics. Results were described using the four constructs of NPT: 1) *Coherence*: Clinicians understood the rationale behind CRISP, particularly since they were familiar with using risk tools for other conditions; 2) *Cognitive participation:* GPs welcomed the opportunity CRISP provided to discuss healthy and unhealthy behaviors with their patients, but many GPs challenged the screening recommendation generated by CRISP; 3) *Collective Action:* CRISP disrupted clinician-patient flow if the GP was less comfortable with computers. GP consultation time was a major implementation barrier and overall consensus was that PNs have more capacity and time to use CRISP effectively; 4) *Reflexive monitoring:* Limited systematic monitoring of new interventions is a potential barrier to the sustainable embedding of CRISP.

**Conclusions:**

CRISP has the potential to improve risk-appropriate CRC screening in primary care but was considered more likely to be successfully implemented as a nurse-led intervention.

## Background

Australia and New Zealand have the highest rates of colorectal cancer (CRC) globally, with estimated age standardized rates of 44.8 and 32.2 per 100,000 in men and women respectively [1]. Risk-stratified screening is increasingly recognized as an approach to maximize the benefits and reduce the potential harms of cancer screening, especially in the era of personalized/precision medicine [2]. In Australia, the National Health and Medical Research Council (NHMRC) guidelines recommend the non-invasive and inexpensive fecal occult blood test (FOBT), using the fecal immunochemical test (FIT), for screening in people at average risk of CRC, and more invasive and expensive colonoscopy only for those who are at increased risk, with risk-stratification based on age and family history criteria [3]. However, it is estimated that for every 1,000,000 Australians aged 50 years and older, 80 000 people at average risk are being over-screened with colonoscopy, 29 000 at increased risk are not having the colonoscopy they need [4, 5], and only 37% participate in the National Bowel Cancer Screening Program (NBCSP) [6]. Over-screening potentially results in excess costs and excess risks to the patient due to the invasive nature of colonoscopy, and under-screening potentially results in missing early-stage cancer or precancerous lesions.

Risk-stratified screening requires valid risk prediction models that accurately discriminate people at increased risk from those who are not. These risk models require easy-to-use risk assessment tools for implementation into clinical practice [7]. A recent systematic review of trials of cancer risk assessment tools in primary care found that few trials have been conducted with risk tools in primary care, and of those trials, many have been limited by methodological flaws including low recruitment rates and a ‘healthy volunteers’ effect [8]. Nevertheless, the review showed that such tools can improve patient risk perception, cancer knowledge and screening intentions, although the effect on actual screening behaviours is less clear. Few risk assessment tools in these trials implemented validated cancer risk models or applied the latest evidence on risk communication methods. Furthermore, several had undergone limited clinical evaluation prior to testing in a full scale randomized controlled trial (RCT), contrary to recommendations about the development and evaluation of complex interventions [9, 10].

Our NHMRC funded ‘Centre for Research Excellence to optimise screening for colorectal cancer’ is undertaking a program of research to develop an Australian CRC risk prediction model, and to implement it into a risk assessment tool for use in primary care (‘general practice’). This paper describes an initial exploratory study based on the UK’s *Medical Research Council Framework for the Development and Evaluation of Complex Interventions* to improve healthcare [9, 10]. This qualitative Phase I study aimed to examine the useability and acceptability of a prototype tool ‘CRISP’ (Colorectal cancer RISk Prediction tool), identify barriers and enablers to implementing CRISP in Australian general practice, and optimize the design of CRISP as an important step in the development of an intervention prior to an efficacy (Phase II) RCT [10, 11].

## Methods

### Study participants

We recruited general practices from a broad range of clinics including some from the Victorian Primary Care Practice-Based Research Network (VicReN). Practice managers (PMs) and practice nurses (PNs) were recruited from the same practices to explore the context for practice implementation. Participants were reimbursed for their time.

### CRISP development

The CRISP tool was programmed as Java software leveraging the Apache Wicket open source framework for web application development. This software tool was designed to implement any epidemiological risk model with simple modifications to data entry fields. Based on a review of previously validated risk models [12], the ‘Freedman’ CRC risk model (with minor modifications) was chosen to be the epidemiological model underpinning the prototype CRISP tool [13].

This study used an ‘action design’ method to allow for changes in CRISP to explore the potential utility of the different iterations of the tool as respondents’ data were analyzed.

### CRC risk assessment

The CRISP risk assessment tool was designed to assist clinicians to collect risk information from patients and provide screening recommendations during a consultation. The CRISP interface was based on the results of our systematic review of cancer risk assessment tools in primary care [8] and previous experience in developing cancer risk tools for use in primary care [14]. Risk variables captured by CRISP included: age, gender, BMI, smoking, exercise, diet, previous colonoscopy, previous polyp, use of non-steroidal anti-inflammatory drugs and family history and age of onset of CRC, ovarian or endometrial cancer, for first and second degree relatives.

CRISP risk outputs included a recommendation for the mode of CRC screening based on five-year absolute CRC risk and a range of visual representations based on current best evidence about risk communication research [15–17], including: (see Figures)

1. a statement of absolute CRC risk over different timeframes using percentages and odds (Fig. 1),

**Fig. 1:**
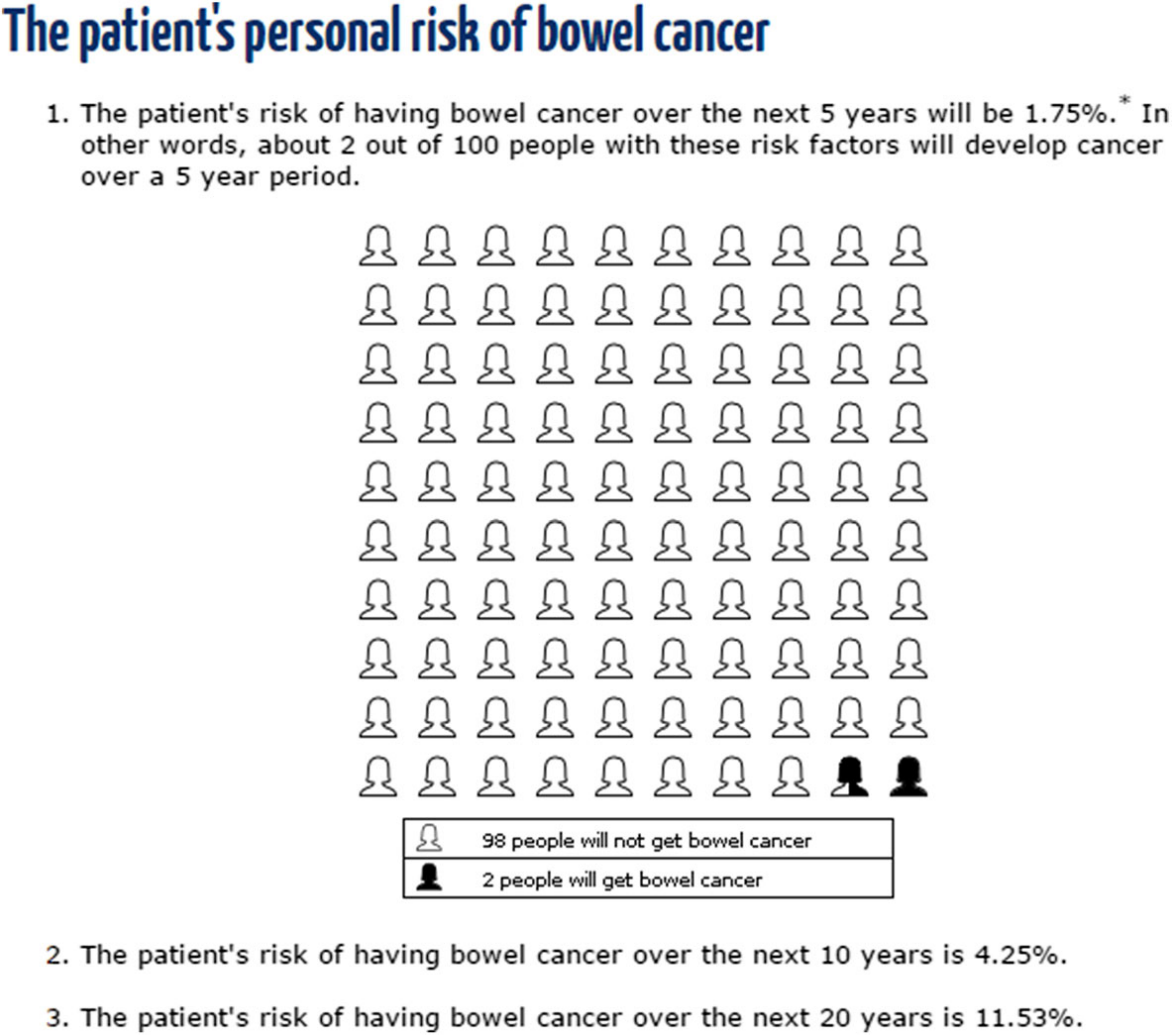
An example of the risk presentation using percentages, odds and a natural frequency icon array produced by the CRISP prototype
2. an icon array demonstrating natural frequencies of 5-year CRC risk (Fig. 1), and
3. a graph showing individual CRC risks over time compared to the average population (Fig. 2).

**Fig. 2:**
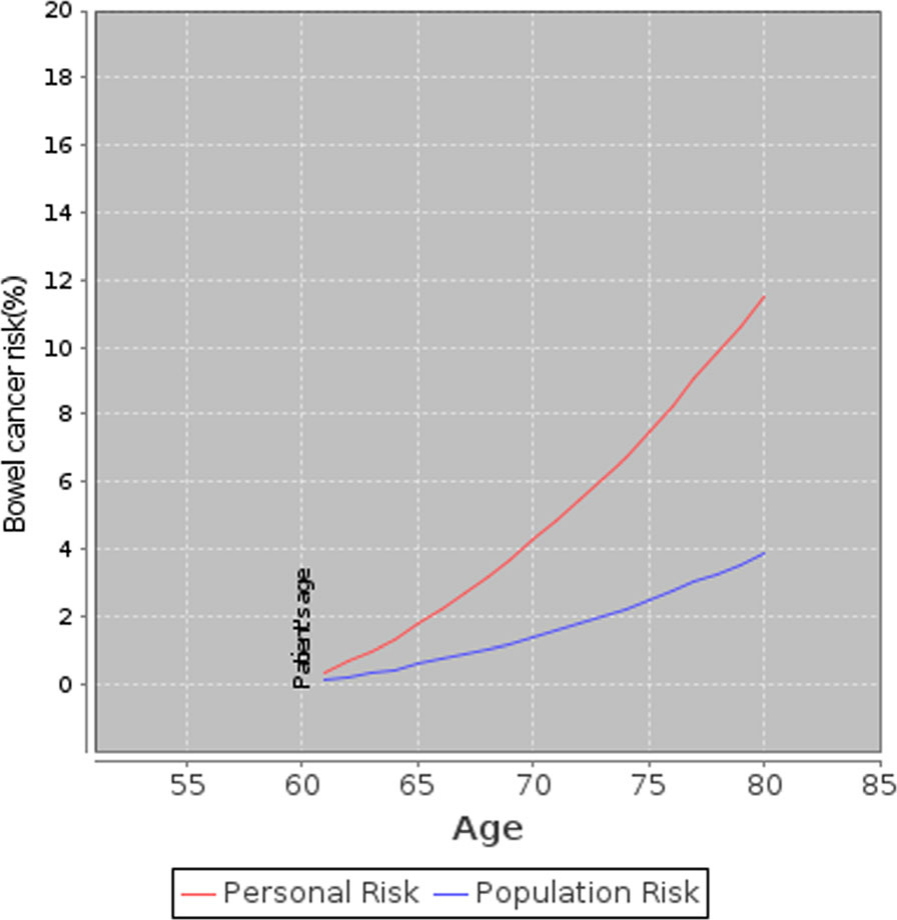
An example of the risk presentation using a graph produced by the CRISP prototype showing a person’s risk and the background population risk of CRC over time

### Clinical recommendations

**Fig. 3:**
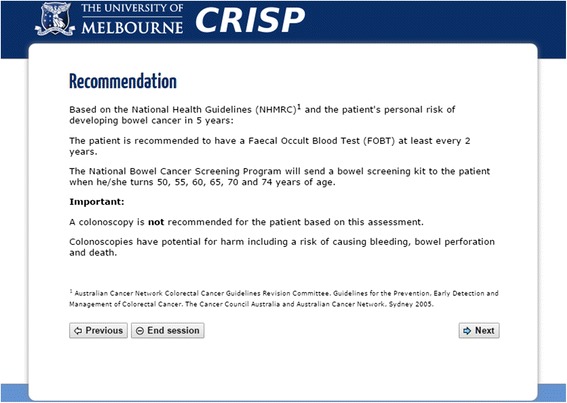
An example of the colorectal cancer screening recommendations produced by CRISP

Based on the NHMRC guidelines [3] and five-year absolute CRC risk, CRISP provided four possible clinical recommendations including: (Fig. 3)

1. no screening for people less than 50 years old with a five-year absolute CRC risk less than 1.00%;
2. FOBT for people 50 years or older with a five-year absolute CRC risk less than 1.00%;
3. colonoscopy for people with a five-year absolute CRC risk greater than or equal to 1.00%; or
4. referral to a familial cancer centre for further risk assessment for people with a strong family history of CRC or family history suggestive of Lynch syndrome.

### Procedures

GPs were familiarized with CRISP using paper-based cases and then conducted two simulated consultations with actors professionally trained to be patients [full descriptions of the case vignettes available in Appendix A]. The actors’ performances were monitored to ensure standardization. The simulated consultations were conducted in the GPs’ consulting rooms and video recorded.

CRISP was demonstrated and explained to the PNs and practice managers PMs.

Semi-structured interviews were conducted by the same interviewer (JGW) after the simulated consultations or CRISP demonstration. Interviews were audio-recorded and transcribed.

The interview guide was developed using Normalization Process Theory (NPT). NPT is a well-established sociological theory which proposes a working model for implementing, embedding and sustaining practices and/or interventions into clinical practice, in particular, in primary care [18]. Acceptability, usability and implementation strategies were explored at an individual level (GP, PN and PM) and organizational level (the practice) using the four domains of NPT: ‘Coherence’, ‘Cognitive participation’, ‘Collective action’ and ‘Reflexive monitoring’ [19].

Data saturation was reached when no new themes were occurring after three consecutive interviews for any of the three groups.

### Analysis

Analysis commenced soon after data collection started. Video footage of the simulated consultations was analyzed to explore important non-verbal observations such as body language of the GP and ‘patient’ and any impact using CRISP had on doctor-patient interaction. The time spent per screen and time to complete a CRISP session as well as any user error were captured electronically. Video footage and audio transcripts were coded in *dedoose 6.0.24®* [20]. A coding framework was developed inductively involving discussions between the authors (JGW, JDE, FMW). The themes that arose from the analysis were then mapped onto the four constructs of NPT. [19] CRISP was modified during the study in response to observations and early data analysis. The revised version of CRISP was reviewed by two GPs who had been previously involved in the study and two GPs unfamiliar with CRISP.

### Ethics approval and consent to participate

All participants were recruited following an informed consent process involving approved plain language statements and consent forms (HREC reference number: 1340491).

## Results

### Sample

We recruited 14 GPs, nine PNs and six PMs from 12 general practices. The characteristics of the participants are described in Table 1.

**Table 1:** Characteristics of general practitioners, practice nurses and practice managers participating in the study

Participants	Characteristics	n (%)^a^
General practitioners		
	Age, years^b^	50 (29, 62)
	Gender, female	7 (50.0)
	Practice location, metropolitan	10 (71.4)
	Number of years in general practice^b^	22 (1, 34)
	Number of GPs working in practice:^b^	
	Full-time	2 (0, 7)
	Part-time	5 (0, 14)
	Hours worked in an average week^b^	30 (12, 50)
	Postgraduate qualifications, yes	7 (50.0)
	More than one qualification	5 (71.4)
Practice nurses		
	Age, years^b^	55 (48, 68)
	Gender, female	9 (100.0)
	Practice location, metropolitan	5 (55.6)
	Number of years in general practice^b^	35 (9, 40)
	Hours worked in an average week^b^	36 (30, 40)
	Specialist qualifications, yes	7 (77.8)
	More than one specialization	5 (71.4)
Practice managers		
	Age, years^b^	50 (29, 58)
	Gender, female	6 (100.0)
	Practice location, metropolitan	4 (66.7)
	Number of years in general practice^b^	2 (1, 21)
	Hours worked in an average week^b^	40 (31, 46)
	Specialist qualifications, yes	2 (33.3)

^a^Unless specified otherwise; ^b^Median (range)

### Results of the analysis using the four constructs of NPT

#### Coherence

‘Coherence’ refers to how the participants make sense of and value an intervention. Clinic staff agreed CRC screening was relevant to their practice and were familiar with using risk assessment tools to support clinical decision making, particularly for assessing cardiovascular or diabetes risk, or when screening for depression.

*So we use quite a few assessment tools in the practice and … obviously find them valuable. So, from that point of view, we’re not unfamiliar with using them and certainly wouldn’t be averse to using them across the board.* [PM: 49 years, female]

GPs used the icon array to explain CRC risk, either to emphasize the need to be screened,

*…but don’t forget that little person; okay let’s make sure that’s not you*. [GP: 50 years, female]

or to reassure the patient that their risk was low.

…*there’s a whole lot of people and this is your chance out of them of cancer*… [GP: 31 years, female]

The graph was more challenging to explain. In the first iteration of CRISP the y axis (CRC risk) ranged from 0% to 20% which generated a steep curve at times, and caused concern that this might mislead patients about their risk (Fig. 3). All staff preferred the natural frequency icon array to the graph depicting comparative CRC risk over time.

Screening advice was challenged by some GPs who did not agree with or were not familiar with current NHMRC guidelines.

*…that’s just the tricky bit of saying* [to the patient] *that this is what’s* [sic] *the recommendation but we don’t always follow what recommendations are*… [GP: 50 years, female]

Many GPs also challenged the necessity for collecting second degree relative information.

*Because once you go beyond that first degree relative, really the risk diminishes so significantly that getting all that extra data isn’t going to be that significant*. [GP: 50 years, female]

### Cognitive participation

‘Cognitive participation’ explores how users engage with and understand an intervention in the context of their everyday work. There were specific aspects of CRISP that everyone liked, including the opportunity to reinforce healthy behaviours or discuss strategies with patients for changing unhealthy behaviours (smoking, diet, and exercise in particular).

*Anything that gets people to have a think about their health is a good thing, anything at all.* [PN: 48 years, female]

The GPs responded easily and almost instinctively to patients’ responses to these questions.

GP: *Do you smoke?*Patient: *No.*GP: *Good man!* [GP 53 years, male]

On the other hand, there was a general distrust of some of the recommendations, particularly for FOBT. Colonoscopy was commonly referred to as the ‘gold standard’ for CRC screening. GPs were more likely to recommend colonoscopy if the patient wanted one or they had previously been recommended one by a gastroenterologist, even if the patient was at average risk.

### Collective action

‘Collective action’ refers to the capacity for the intervention to fit within existing organizational practices. GPs, PNs and PMs all agreed that lack of GP consultation time would limit the use of CRISP by GPs. There was a consensus that nurses have the capacity, time and expertise to complete the risk assessment as part of routine preventive health consultations.

*… nurses … see the patient first a lot of the time and so they could go through these sorts of things…* [PM: 48 years, female]

The existing health checks for 45 – 49 year olds [21] which are funded by the Australian government were suggested as an optimum time for PNs to introduce CRISP.

Opinions about who would take responsibility for the final decision about screening advice was split between GPs and PNs; many GPs had a fear of missing a diagnosis and they often relayed anecdotes of cases of a missed or late CRC diagnosis.

… *I’ve got a 26-year-old patient that has …metastases everywhere …of course, everybody said it was her endometriosis*… [GP: 50 years, female]

Using CRISP as a self-completed tool in the waiting room was explored but everyone felt that administrative staff would not have the time or expertise to manage patients using CRISP in the waiting room.

*…Yeah. I just don’t think it will work in the waiting room…We might start getting asked questions at the front desk… That’s the thing, how do we answer? You know what I mean?* [PM: 29 years, female]

Implementation of new interventions into a practice was generally the GPs’ decision although PMs and PNs felt they could propose new ideas to the practice.

*…the doctors are very open to suggestion. If the practice nurses, management, or another doctor feels that this could be a really good idea for the doctors, it’s taken to the doctors’ meeting and overall feeling of shall we do this, shall we not is taken place…*[PM: 42 years, female]

Despite this, even if a decision was made at a practice level, GPs still acted independently when incorporating new technology into their clinical work.

*So I’m in a really big group practice… and … people would be really excited…* [But it] *would vary because some people don’t change their practice much at all…* [GP: 33 years, female]

Many GPs found certain aspects of using the CRISP prototype laborious, especially the method for collecting family history information.

*…this is a labyrinth!… especially having 3 pages for the 2nd degree relatives…*[GP: 50 years, female]

GPs wanted CRISP integrated into existing clinical software so data could be auto-populated and then updated into the patient record but acknowledged that the variety of software used in different practices would make it difficult to integrate CRISP universally.

There were challenges using CRISP within a GP consultation. GPs felt uncomfortable taking their attention away from the patient by looking at the computer. Despite this, GPs often re-engaged with the patient when explaining the risk output and screening recommendations. Younger GPs, particularly those who could touch type, were able to move more seamlessly between the computer and the patient. When GPs asked questions of their patients prior to opening CRISP, which was not uncommon, they felt the tool was unnecessarily repetitive.

### Reflexive monitoring

‘Reflexive monitoring’ refers to how new interventions are monitored, evaluated and appraised [19]. Clinics evaluated their practice in an ad hoc way either focusing attention on a particular ‘disease of the month’, or because a PN or PM had a special interest in a clinical condition or in practice management. It was unusual for a clinic to have regular audits as part of ongoing quality assurance. Time and poor coding of data were two main obstacles to evaluating clinical practices.

… *the capacity’s there. Yeah, and we certainly have things that we can do like that, but often we do nothing. … - it’s like ships in the night, so we can be here and not even say hi to each other all day sometimes*…[PM: 55 years, female]

Consequently, there were limited systems within the practices to monitor or evaluate the effectiveness of CRISP over time.

## Discussion

### Main findings

As a preliminary qualitative study in a program of research to develop and trial a novel CRC risk prediction tool, we identified clear barriers and facilitators to the implementation of our prototype tool (CRISP) into general practice. We found that GPs were capable of using CRISP in simulated consultations, and were generally used to applying risk tools as part of their clinical decision making. Despite this, GPs did not always adhere to the CRISP screening advice, with many favouring colonoscopic screening even for people at average risk as it was considered a ‘better’ screening method. Barriers to using CRISP included limited consultation time, long-winded family history collection, and for some GPs, lack of confidence with computers. All clinic staff thought the questions about lifestyle were very helpful for facilitating a discussion about how to change unhealthy behaviors and could see how CRISP might fit into a health-check conducted by the PN. For CRISP to be implemented into practice, it was obvious that it would need to be more intuitive and shorter and would be easier if it was integrated into the patient clinical medical record.

### Iterative modifications to the CRISP tool during the study

To increase the useability of CRISP, we reduced the data entry pages from up to 27 pages to a maximum of eight pages. Other modifications included:

1. development of an interactive family history collection page on a single web-page to collect family history; this was more intuitive and user friendly, and substantially reduced the time to complete CRISP (Fig. 4);

**Fig. 4:**
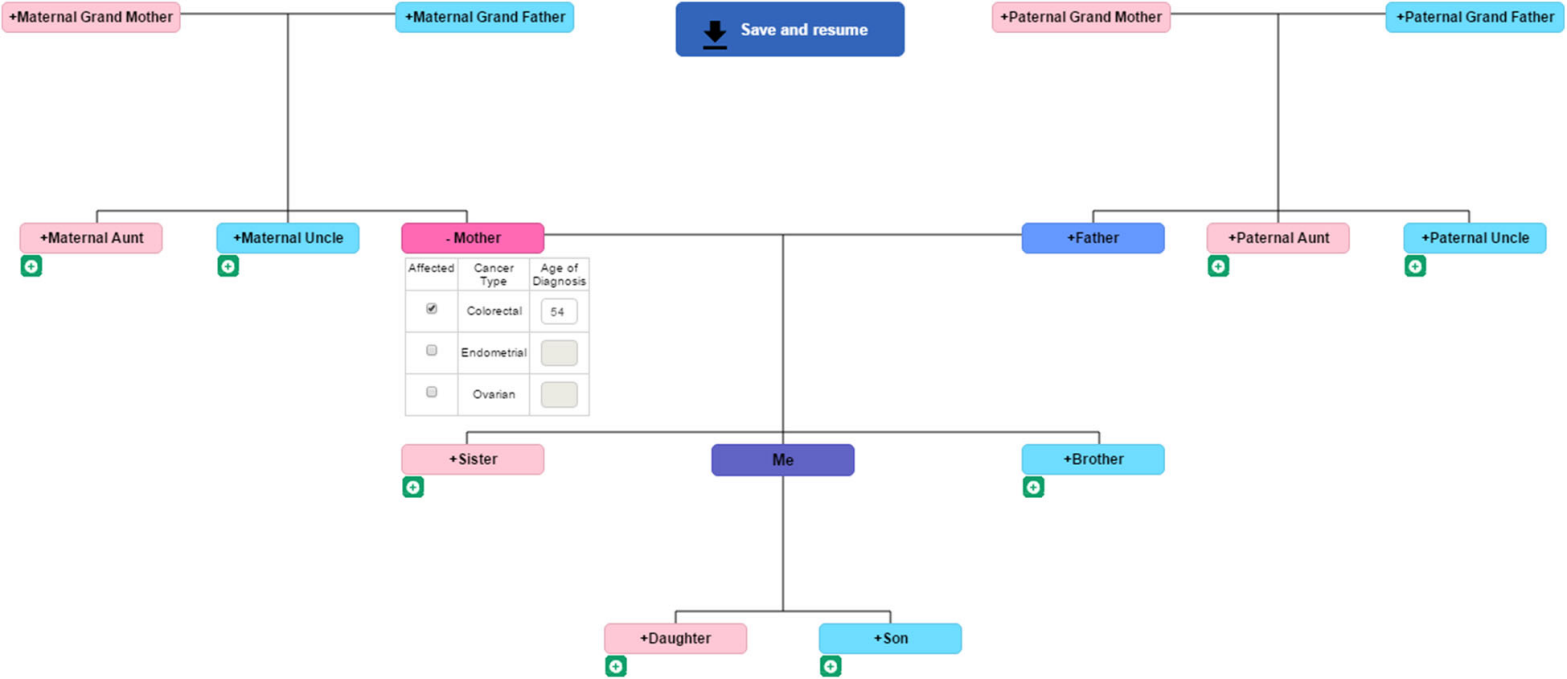
The revised interactive family history collection page to capture family history of cancer in CRISP
2. modifications to the natural frequency icon array to include a larger denominator population and therefore present smaller risks more clearly (Fig. 5);

**Fig. 5:**
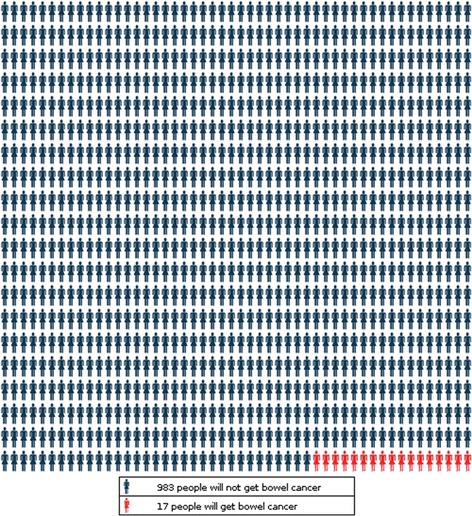
An example of the revised icon array in the CRISP tool to demonstrate absolute risk of colorectal cancer over 5 years per 1000 people
3. modifications to the graph to reduce potential overestimation of risk over time (Fig. 6); and

**Fig. 6:**
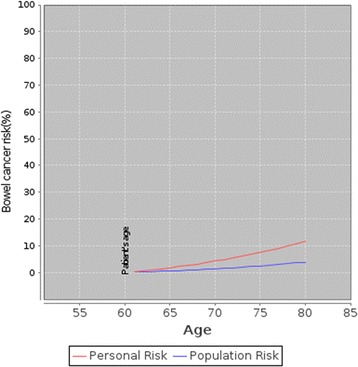
An example of the revised graph presented by CRISP to illustrate absolute risk of colorectal cancer over time relative to the average risk
4. as a result of our findings on GPs’ perceptions about the relative benefits and harms of FOBT and colonoscopy, we developed additional risk communication output in CRISP to highlight the potential harms of colonoscopy and the sensitivity of FOBT to detect CRC using ‘expected frequency trees’ (Fig. 7) [16].

**Fig. 7:**
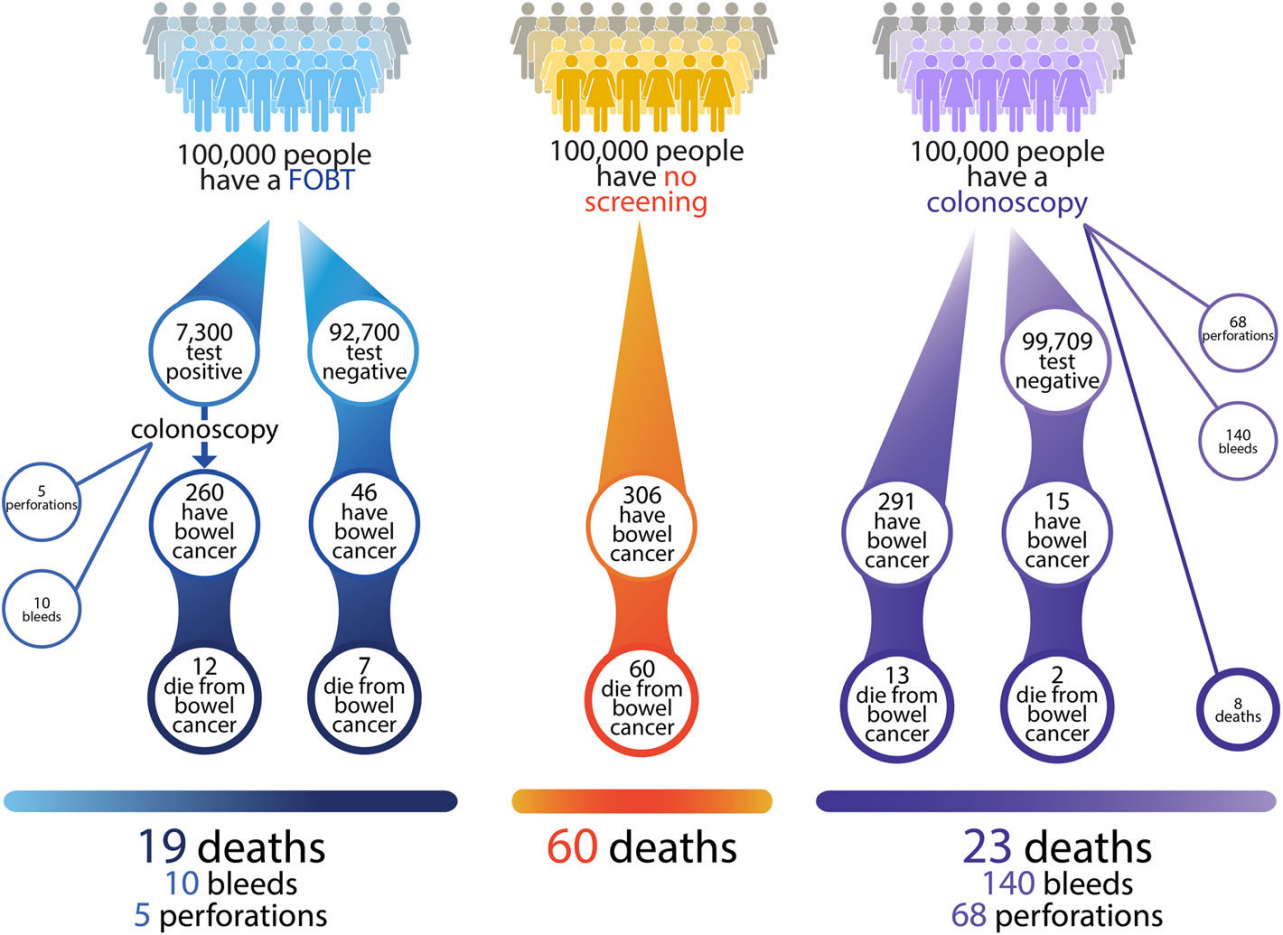
Expected frequency tree demonstrating the benefits and harms of FOBT and colonoscopy screening for 100 000 patients at average risk

### Context

Our results are consistent with findings about the few available cancer risk assessment tools used in primary care [8]. The use of a dedicated clinician such as a PN has the potential to increase uptake of the risk tool [22, 23] and, similar to findings in the *Family Healthware Impact* Trial, health promotion within a risk tool increased clinician ‘buy in’ [24]. In comparison with other risk tools, GPs did not experience a sense of ‘loss of control’ of the consultation when using the tool, even if the screening advice contradicted their expectations [23, 25].

The CRISP research team previously conducted an exploratory study of a diagnostic risk assessment tool (QCancer) using similar methods, but the CRISP study involved GPs, PNs and PMs. CRISP was more acceptable to GPs than QCancer, although both were difficult to introduce into a GP consultation. The advantage of interviewing all staff in the CRISP study was the discovery that clinic staff were in agreement that CRISP would be better suited to use in a nurse consultation. Another significant difference between QCancer and CRISP was the user interface. A major limitation to the accuracy of QCancer was input error [25]. This is a common limitation to the accuracy of medical technology in clinical care [26], but was not found to be a factor in this study. Perhaps more significantly though was the nature of the clinical task being supported by the risk assessment tool. GPs in this study were less confronted by discussing future risks of colorectal cancer than they were using the QCancer tool which presented risks of current undiagnosed cancer for multiple tumour types.

Our study demonstrated that primary care in Australia has the capacity to implement risk assessment tools using PNs and potentially contribute to more risk-appropriate CRC screening. Practice staff felt CRISP would fit into the context of a preventive health consultation conducted by a PN, in particular as part of ‘45 – 49 year old’ health checks already funded by the Australian government to increase screening for chronic diseases [21]. This provides the opportunity to prime patients to participate in the NBCSP prior to receiving their NBCSP FOBT after turning 50, and identify patients at higher risk of CRC for whom colonoscopy and/or genetic counselling may be indicated. The variety of risk communication formats employed in the CRISP tool is designed specifically to support discussion and shared decision making where there is a choice of screening test and different harms and benefits to consider. The expected frequency trees are expected to challenge GPs’ beliefs about the superiority of colonoscopic screening. Implementing CRISP as a PN-led intervention has the advantage of nesting CRISP into an existing model of care, which frequently involves shared decision making about disease prevention. This will also minimize the impact on GPs’ limited consultation time.

### Strengths and limitations

Although some of the participants were recruited from a practice-based research network, we recruited a range of GPs, PNs and PMs including: 1) rural and metropolitan practice staff, 2) solo and group practice staff, and 3) staff from a range of ages and experience level in general practice. The sample of PNs was mainly female and of similar ages, however, this was not atypical for this population and is likely to be representative [27]. This applied to PMs as well.

We attempted to recruit GPs, PNs and PMs from the same clinics, to assess congruency of ideas between staff at the same clinic. However, this was not always possible with some clinic staff unavailable for an interview and one clinic did not have a PN.

Another limitation was the potential for social desirability bias as participants might not have felt comfortable criticizing CRISP to the researchers during the interviews. This was offset by the data gained from the simulated consultations which have been demonstrated to predict actual clinical performance [28].

## Conclusions

This study has provided important contextual information on some of the potential barriers and facilitators to implementing CRISP and risk-stratified CRC screening in primary care. It has informed further development of CRISP so that it is more likely to meet the needs of users and provide risk information that could support improved screening decision making. We have identified a preferred model of implementation in practice involving PNs. This approach will now be tested in an RCT to assess the effects of implementing CRISP on GP and patient CRC screening behaviours.

## Appendix A

### Actors’ Vignettes

**Case 1:**

‘Barry Green’ is attending the clinic for the first time as a new patient. He has just moved to Melbourne from interstate and wants to organize to have another colonoscopy as he had one a ‘few years ago’ because his mother had bowel cancer (diagnosed at 58). Barry’s previous colonoscopy showed no sign of any polyps or bowel disease.

Barry is a 48 year old male and is 178 cm tall and weighs 80kgs (BMI of 25). He has no current bowel symptoms and no previous diagnosis of colorectal cancer.

He never uses non-steroidal anti-inflammatories (NSAIDS), eats vegetables 3 – 4 times per week and exercises for up to 3 h per week. He doesn’t smoke.

His family history: Mother diagnosed with CRC at 58 years old (deceased).

Unaffected relatives include: father, 1 brother, 1 sister, 2 daughters, 2 paternal aunts, 1 niece, 1 nephew.

**Case 2:**

‘Glenda Brown’ is an office worker aged 57. She is 170 cm and weighs 80kgs (BMI: 27.7). Glenda has come to see her GP for a check-up as she has been feeling tired lately.

She is ‘fairly healthy’ and doesn’t have any obvious symptoms. She stopped menstruating a couple of years ago but she has not taken hormone replacement therapy as she has had no problems with menopause.

She works in an office and does no vigorous exercise. She eats well — including eating vegetables every day (7 days a week).

When asked about family history: Her brother has been diagnosed with bowel cancer (aged 54) and her mother died from bowel cancer as well and she was also diagnosed in her mid-fifties (55). She has never had a colonoscopy and not done a Faecal Occult Blood Test (FOBT) partly because she is a bit worried.

She has smoked cigarettes since she was 25 (15 cigarettes per day) which she has trouble giving up.

She doesn’t take any NSAIDS regularly.

Unaffected relatives include: parents, grandparents, one sister, one paternal uncle (father’s brother) and one niece.

## Abbreviations

CRC: Colorectal cancer
CRISP: Colorectal cancer RISk prediction tool
FIT: Fecal immunochemical test
FOBT: Fecal occult blood test
GP: General practitioner
NHMRC: National health and medical research council (Australia)
NPT: Normalization process theory
PM: Practice manager
PN: Practice nurse
RCT: Randomized controlled trial
